# Frailty in Preclinical Models: Impact of Healthspan on Disease Expression Over the Life Course

**DOI:** 10.1093/geroni/igab046.120

**Published:** 2021-12-17

**Authors:** Susan Howlett

**Affiliations:** Dalhousie University, Halifax, Nova Scotia, Canada

## Abstract

People age at different rates. This heterogeneity in aging has led to the concept of “frailty”, a state of heightened vulnerability to adverse health outcomes at any age. Frailty challenges health care providers, as frail patients are more likely than non-frail patients to experience diseases, hospitalization, and death. We showed that frailty occurs not only in humans, but also in aging rodents. It can be measured with a “frailty index” (FI) based on age-related health deficit accumulation as originally established in humans. We found that maladaptive changes in heart structure and function in late life are correlated more so with frailty than age and are closely graded by FI score, especially in male mice. Adverse effects of frailty originate at cellular/subcellular levels and scale up to organ and system levels, predisposing towards cardiovascular disease. Poor overall health, quantified with an FI, may drive maladaptive cardiac remodeling, especially in older males.

